# The Hypothalamic Medial Preoptic Area–Paraventricular Nucleus Circuit Modulates Depressive-Like Behaviors in a Mouse Model of Postpartum Depression

**DOI:** 10.34133/research.0701

**Published:** 2025-05-14

**Authors:** Ping Fu, Cui-Ping Liu, Cheng-Yi Liu, Yan-Chu-Fei Zhang, Ju-Ping Xu, Rui-Ting Mao, Xue-Ying Ding, Fan Li, Yi-Long Zhang, Hai-Long Yang, Jing-Ning Zhu, Guo Zhang, Jian Jing

**Affiliations:** ^1^ Department of Neurology and Medical Psychology, Nanjing Drum Tower Hospital, State Key Laboratory of Pharmaceutical Biotechnology, Institute for Brain Sciences, Chinese Academy of Medical Sciences Research Unit of Extracellular RNA, Jiangsu Engineering Research Center for MicroRNA Biology and Biotechnology, Chemistry and Biomedicine Innovation Center, School of Life Sciences, Nanjing University, Nanjing, Jiangsu, China.; ^2^ Department of Medical Psychology, Nanjing Drum Tower Hospital, The Affiliated Hospital of Nanjing University Medical School, Nanjing, China.; ^3^ Department of Neuroscience and Friedman Brain Institute, Icahn School of Medicine at Mount Sinai, New York, NY, USA.; ^4^ Peng Cheng Laboratory, Shenzhen, China.

## Abstract

Estrogen fluctuations have been implicated in various mood disorders, including perimenopausal and postpartum depression (PPD), likely through complex neural networks. γ-aminobutyric acid-ergic (GABAergic) neurons in the medial preoptic area (MPOA) that express estrogen receptor 1 (ESR1) are essential for the development and expression of depressive-like behaviors in ovarian hormone withdrawal (HW) mice. However, the precise circuit mechanisms through which MPOA GABAergic neurons influence behavior remain incompletely understood. Here, we identified robust projections from MPOA GABAergic neurons to the paraventricular nucleus of the hypothalamus (PVN). In HW mice, chemogenetic activation of MPOA GABAergic neurons targeting PVN attenuated depressive-like behaviors. Conversely, in nonhormone withdrawal (NHW) control mice (which received continuous estrogen), suppression of MPOA GABAergic projections to PVN exacerbated depressive-like behaviors. Further analyses using quantitative polymerase chain reaction and immunostaining identified arginine vasopressin (AVP) as a key neuropeptide in this pathway in the HW mouse model. Chemogenetic inhibition of PVN^AVP^ neurons significantly alleviated depressive-like behaviors in HW mice, while their activation in NHW mice worsened depressive-like behaviors. These behaviors were dependent on AVP expression in PVN^AVP^ neurons. Moreover, in HW mice, chemogenetic inhibition of PVN^AVP^ neurons receiving MPOA input mitigated depressive-like behaviors. Conversely, in NHW mice, activation of these neurons exacerbated depressive-like behaviors. Electrophysiological recordings demonstrated that MPOA GABAergic neurons directly inhibit PVN^AVP^ neurons. Thus, our findings suggest that PVN^AVP^ neurons serve as downstream effectors of MPOA GABAergic neurons via monosynaptic inhibitory signaling to regulate depressive-like behaviors. Targeting this circuit may offer a novel therapeutic strategy for PPD.

## Introduction

Reproductive hormone levels, particularly the fluctuations following childbirth, influence a range of physiological, behavioral, and emotional states [1]. For instance, estrogen, which is primarily synthesized in the placenta, increases by 100- to 1,000-fold during late pregnancy and then rapidly declines to pre-pregnancy levels postpartum [2]. Significant fluctuations in estrogen levels are closely linked to a heightened risk of developing postpartum depression (PPD), characterized by symptoms such as anhedonia, helplessness, social impairment, and anxiety [1,3–5]. These symptoms are thought to arise from complex neural circuits distributed throughout the brain.

Research has identified several brain regions with high estrogen receptor expression that may detect changes in reproductive hormone levels, including the hippocampus [6–8], amygdala [8], and medial preoptic area (MPOA) [9]. The MPOA, a sexually dimorphic structure with particularly high estrogen receptor 1 (ESR1) expression [9–11] in females, appears to be a central site for integrating information about estrogen level fluctuations. The MPOA contains both glutamatergic and γ-aminobutyric acid-ergic (GABAergic) neurons and is essential in modulating social behaviors, such as parenting [12–18], mating [16,19], and social interactions [20–22]. Additionally, it regulates basic physiological processes, including body temperature [23], sleep [24], and feeding [25,26]. Relevant to postpartum mood disorders, a recent study using a hormone withdrawal (HW) mouse model has identified MPOA GABAergic neurons, particularly those expressing ESR1, as crucial in modulating depressive-like and anxiety-like behaviors [9]. Specifically, projections from MPOA^GABA^ neurons to the ventral tegmental area (VTA) regulate anhedonia, while projections to the periaqueductal gray (PAG) mediate immobility [9]. Although this study established the importance of the circuits from MPOA^GABA^ projecting to VTA/PAG for depression in HW mice, whether and how MPOA^GABA^ neurons could target other brain regions remains unexplored.

We sought to study the potential role of MPOA projections to the paraventricular nucleus of the hypothalamus (PVN) in an HW mouse model. Previous studies have implicated that the PVN is a potential contributor to the development and expression of depressive-like behaviors [27–29]. For example, in the chronic restraint stress (CRS) mouse model, chemogenetic activation of PVN neurons has been shown to exacerbate depressive-like behaviors, while their inhibition attenuates these behaviors [29]. Research on maternal and infanticidal behaviors has also shown that MPOA^ESR1^ neurons project to the PVN with a notably high density of these projections in female mice [21,30], and a reduction in MPOA GABAergic neuronal activity has been observed in the HW models [9]. These results led us to propose that the MPOA^GABA^-PVN circuit might be involved in mediating depressive-like behaviors triggered by the fluctuations of reproductive hormones.

Within the PVN, arginine vasopressin (AVP) neurons represent a critical population involved in regulating various physiological and behavioral functions, including sleep, feeding, self-grooming, nest building, social investigation, and aggression [31–35]. Moreover, PVN^AVP^ neurons are also implicated in stress responses, with previous research describing increased AVP-positive neurons in the PVN of depressed patients and rats [36,37]. Genetic ablation of AVP has been shown to enhance sucrose preference and reduce immobility time in forced swimming test (FST) [38], indicating a potential role in modulating depressive-like behaviors. Notably, a clinical study has implicated AVP as a potential contributor to PPD [39]. Despite these insights, the specific circuit mechanisms by which AVP regulates depressive-like behaviors, particularly those related to reproductive hormone fluctuations, remain largely unexplored.

Our study demonstrates that in HW mice, activation of MPOA GABAergic neurons projecting to PVN^AVP^ neurons alleviates depressive-like behaviors. Conversely, in nonhormone withdrawal (NHW) mice, suppression of these MPOA GABAergic neurons targeting PVN^AVP^ neurons exacerbates depressive-like behaviors. Furthermore, chemogenetic suppression of PVN^AVP^ neurons in HW mice mitigates depressive-like behaviors, while activation of these neurons in NHW mice worsens these behaviors. Electrophysiological recordings reveal that MPOA GABAergic neurons directly inhibit PVN^AVP^ neurons. Collectively, these findings suggest that MPOA GABAergic neurons exert inhibitory control over PVN^AVP^ neurons in NHW mice, and reduced activity of MPOA GABAergic neurons contributes to depressive-like behaviors in HW mice. This highlights a critical role of MPOA^GABA^-PVN^AVP^ circuit in the pathophysiology of PPD in the mouse model.

## Results

### MPOA GABAergic neurons modulate depressive-like behaviors in HW and NHW mice

To evaluate MPOA ESR1 neuronal activity, we established an ovarian HW model (Fig. S1A). HW mice exhibited depressive-like behaviors (Fig. S1B to G), as indicated by decreased center time in the open-field test (OFT) (Fig. S1B), reduced sucrose preference in the sucrose preference test (SPT) (Fig. S1C), and increased immobility in both the tail suspension test (TST) and forced swim test (FST) (Fig. S1D and E). Additionally, HW mice displayed impaired social discrimination (Fig. S1G), while social preference remained unaffected (Fig. S1F).

The MPOA, a key brain region enriched with ESR1-positive neurons, has been implicated in the regulation of depressive-like behaviors [9]. We performed immunofluorescence that revealed comparable ESR1-positive cell counts between HW and NHW groups (Fig. S1H and I). However, HW mice exhibited reduced c-Fos expression (Fig. S1J) and lower ESR1/c-Fos coexpression (Fig. S1K), indicating diminished ESR1 neuronal activity. Notably, HW mice showed ESR1 labeling primarily in the nuclei, whereas NHW mice exhibited ESR1 localization in the plasma membrane. This membrane localization in NHW mice may be attributed to the palmitoylation of ESR1 following 5 weeks of β-estrogen (EB) treatment, consistent with previous findings demonstrating that EB promotes ESR1 palmitoylation, facilitating its membrane localization [40]. Given that most MPOA ESR1 neurons are GABAergic and MPOA GABAergic neuron activity is reduced in HW mice [9], we focused on MPOA GABAergic neurons in the rest of our study.

Previous optogenetic studies have demonstrated that MPOA GABAergic neurons play a role in depressive behaviors [9]. In this study, we employ chemogenetics to further explore their role. We stereotaxically injected AAV-DIO-hM3Dq-mCherry or AAV-DIO-hM4Di-mCherry into the MPOA GABAergic neurons of GAD2-IRES-Cre mice (Fig. S2A and I) to selectively target GABAergic neurons. Following administration of deschloroclozapine (DCZ), a selective DREADD receptor agonist for hM3Dq (activation) and hM4Di (inhibition), we observed that activation of MPOA GABAergic neurons increased c-Fos expression (Fig. S2B), while inhibition decreased c-Fos expression (Fig. S2J). In HW mice, chemogenetic activation of these neurons alleviated depressive-like behaviors, as evidenced by increased center time in the OFT (Fig. S2C), enhanced sucrose preference (Fig. S2D), and reduced immobility time in the TST and FST (Fig. S2E and F). Additionally, social discrimination improved without affecting social preference (Fig. S2G and H). Conversely, inhibition of MPOA GABAergic neurons in NHW mice exacerbated depressive-like behaviors (Fig. S2K to O). Collectively, these findings, combined with previous optogenetic investigations [9], provide strong evidence supporting the critical role of MPOA GABAergic neurons in regulating depressive-like behaviors in HW mice.

### PVN receives direct inputs from MPOA^GABA^ neurons

**Fig. 1.**
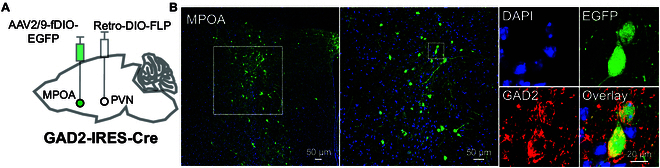
PVN as a critical downstream target of MPOA GABAergic projections. (A) Schematic diagram of virus injection into the MPOA and PVN in female mice. (B) Confocal images of coronal brain sections showing MPOA^GABA^ neurons (green). The middle image is an enlarged view of the area marked by the dashed square in the left image. The right EGFP image further enlarges the middle panel (indicated by the dashed square). Additional images show 4′,6-diamidino-2-phenylindole (DAPI)-stained nuclei (blue), GAD2 (GABA, red), and an overlay of EGFP, DAPI, and GAD2, all in the same field.

Previous studies have identified MPOA neurons expressing ESR1 that project to the PVN [21,30], with a substantial subset of these MPOA neurons being GABAergic [9]. Based on these findings, we hypothesized that MPOA GABAergic neurons may innervate the PVN. To investigate this, rAAV2/R-EF1α-DIO-FLP-WPRE was injected into the PVN, followed by injecting rAAV-hSyn-fDIO-EGFP-WPREs into the MPOA of GAD2-IRES-Cre mice (Fig. 1A). After 3 weeks, retrograde labeling confirmed that MPOA GABAergic neurons project to the PVN (Fig. 1B), suggesting that these neurons may serve as an afferent population to the PVN.

### The MPOA^GABA^-PVN circuit regulates depressive-like behaviors in female mice

**Fig. 2.**
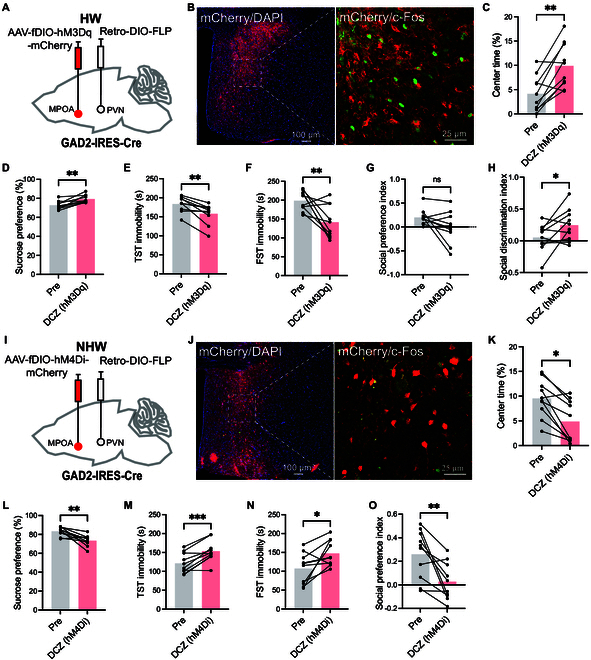
Chemogenetic manipulation of MPOA^GABA^ neurons projecting to the PVN modifies depressive-like behaviors in HW and NHW mice. (A) Schematic of chemogenetic activation of MPOA^GABA^ neurons receiving retrograde input from PVN. (B) Representative images showing virus expression (red) and DAPI (blue) in the MPOA. The right image is an enlargement of the dashed area in the left image. (C to H) Behavioral assessments with or without DCZ: (C) center time in the OFT (**P = 0.0019), (D) sucrose preference in the SPT (**P = 0.0016), (E) immobility in the TST (**P = 0.0021), (F) immobility in the FST (**P = 0.0092), (G) social preference in the 3-chamber test (*P* = 0.0762), and (H) social discrimination in the 3-chamber test (*P = 0.0498). (I) Schematic of viral infection in ovarian NHW mice. (J) Representative images of virus (red) and DAPI (blue) expression in MPOA. (K to O) Behavioral assessments with and without DCZ in NHW mice: (K) center time in the OFT (*P = 0.0172), (L) SPT consumption (**P = 0.0024), (M) TST immobility (***P = 0.0005), (N) FST immobility (*P = 0.0244), and (O) social preference in the three-chamber test (**P = 0.0052). All statistical tests were paired *t* tests (*n* = 10).

To further explore the potential role of the MPOA GABAergic neurons projecting to the PVN in mediating depressive-like behaviors in HW mice, we employed flippase recombinase (FLP)-dependent viral vectors for targeted modulation of this pathway. Specifically, adeno-associated virus (AAV) vectors encoding hM3Dq or hM4Di receptors were bilaterally administered into the MPOA, while Retro-DIO-FLP was injected into the PVN of GAD2-IRES-Cre mice (Fig. 2A, B, I, and J). After 3 weeks, chemogenetic activation of MPOA GABAergic neurons projecting to the PVN increased center time in the OFT (Fig. 2C) and sucrose preference (Fig. 2D), reduced immobility time in the TST and FST (Fig. 2E and F), and improved social discrimination (Fig. 2H) without altering social preference (Fig. 2G) in HW mice. Conversely, chemogenetic inhibition of these neurons in NHW mice exacerbated depressive-like behaviors (Fig. 2K to O). Given that MPOA neurons also project to the PAG/VTA to modulate depressive-like behavior [9], we determined whether MPOA neurons projecting to the PVN and the PAG/VTA represent distinct or overlapping cell populations with retrograde tracing [cholera toxin B subunit (CTB)] in female mice. The results (Fig. S3) showed that although some MPOA neurons project only to the PVN, there are neurons that project both to the PVN and the PAG (Fig. S3D) or the VTA (Fig. S3H), suggesting that MPOA neurons we manipulated in Fig. 2 could act, at least partially, on the PAG or the VTA to modulate behavior. Overall, these results suggest that the population of PVN cells connected to MPOA GABAergic neurons may play a crucial role in mediating the expression of depressive-like behaviors in HW mice.

### PVN^AVP^ neurons respond to ovarian HW

Although the above evidence indicates that activating the MPOA^GABA^-PVN circuit reduced depressive-like behaviors in HW mice (Fig. 2), the specific type of PVN neurons involved is unclear. Because PVN neurons express neuropeptides, we sought to identify neuropeptide signaling involved in depressive-like behaviors within the PVN. First, we examined neuropeptide expression via quantitative polymerase chain reaction in the hypothalamus, which contains the PVN. The HW group exhibited increased expression of oxytocin (OXT) and AVP, suggesting their potential roles in HW-associated depressive-like behaviors (Fig. S4A).

**Fig. 3.**
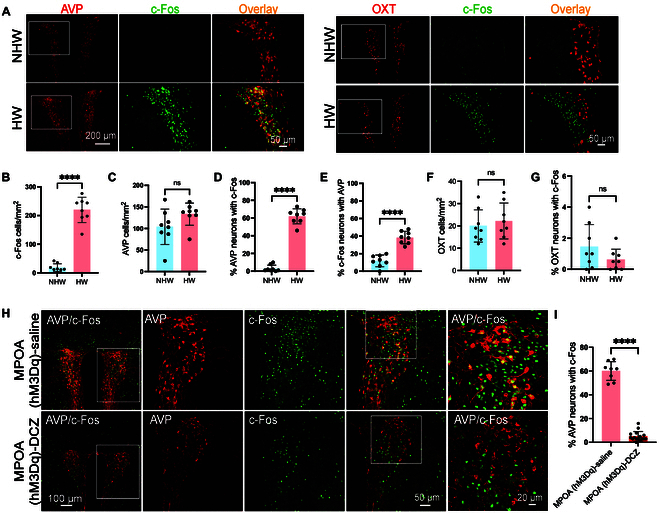
Effects of HW on AVP and OXT-producing cells in HW mice. (A) Representative images showing c-Fos (green) costained with AVP/OXT (red) in PVN. (B) Quantification of c-Fos-positive neurons in PVN (****P < 0.0001, *n* = 5). (C to G) Quantification of AVP/OXT expression: (C) AVP-positive neurons per mm^2^ in PVN (*P* = 0.1088, *n* = 5). (D) Proportion of AVP neurons expressing c-Fos (****P < 0.0001, *n* = 5). (E) Proportion of HW-induced c-Fos neurons expressing AVP (****P < 0.0001, *n* = 5). (F) OXT-positive neurons per mm^2^ (*P* = 0.3570, *n* = 4). (G) Proportion of OXT neurons expressing c-Fos (*P* = 0.1636, *n* = 4). (H) Representative immunofluorescent images of c-Fos (Green) and AVP neurons (red) in the PVN of HW mice after saline (top) or DCZ treatment, i.e., chemogenetic activation of MPOA^GABA^ neurons (bottom). (I) Percentage of c-Fos-positive neurons that coexpress AVP after saline or DCZ treatment in HW mice (*n* = 5, ****P < 0.0001). All statistical comparisons were performed using unpaired *t* tests.

We then utilized immunostaining to examine the activation level of AVP and OXT neurons in conjunction with c-Fos, a marker of neuronal activity, in HW mice. Our findings revealed elevated activation of PVN neurons in HW-treated mice compared to NHW controls (Fig. 3A and B). Notably, a substantial proportion of AVP-expressing neurons exhibited increased activation in the PVN of HW mice (Fig. 3D and E), without a corresponding increase in the total number of AVP-positive neurons compared to NHW mice (Fig. 3C). In contrast, the number of OXT-expressing neurons and the activation proportion of these neurons remained unchanged between the 2 groups (Fig. 3F and G). It is also noteworthy that no significant differences were observed in the activation of AVP and OXT neurons between HW and NHW mice in the supraoptic nucleus (SON) of the hypothalamus where AVP and OXT are known to be highly expressed (Fig. S4B to G). Collectively, these data indicate that the specific activation of PVN^AVP^ neurons, rather than PVN^OXT^ neurons, in the HW mice may contribute to the expression of depressive-like behaviors, suggesting a functional involvement of these neurons in mood regulation.

Furthermore, in HW mice when MPOA GABAergic neurons were not activated by chemogenetics, immunofluorescence analysis revealed that 62.1% of AVP neurons in the PVN coexpressed c-Fos. In contrast, in HW mice when MPOA GABAergic neurons were activated by chemogenetics, the colocalization of AVP with c-Fos in PVN neurons substantially decreased to 5.2% (Fig. 3H and I), suggesting that the MPOA^GABA^ neurons may inhibit PVN^AVP^ neurons. Notably, immunofluorescence staining showed that the PVN lacks ESR1 expression (Fig. S5A). In contrast, the MPOA exhibits robust ESR1 expression (Fig. S5B), consistent with previous studies [30]. These results imply that PVN^AVP^ neurons may be regulated by ESR1-expressing GABAergic neurons in the MPOA, rather than being directly modulated through ESR1. This prompted us to explore the specific contribution of PVN^AVP^ neurons to depressive-like behaviors as described below.

### Chemogenetic manipulation of PVN^AVP^ neurons influences depressive-like behaviors in HW and NHW mice

**Fig. 4.**
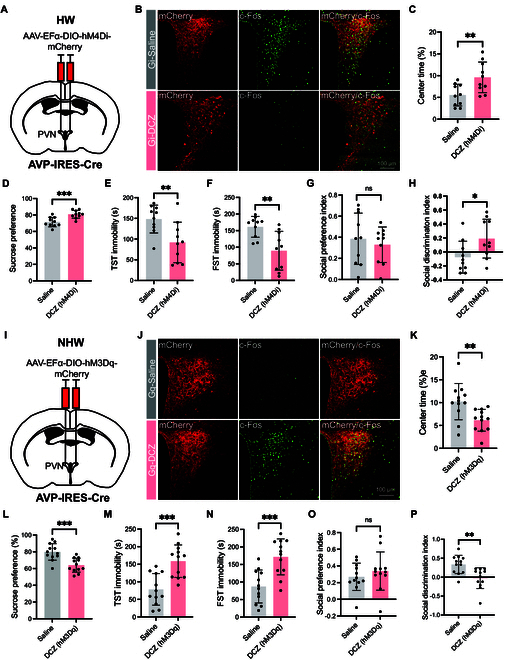
Chemogenetic manipulation of PVN^AVP^ neurons modulates depressive-like behaviors in HW and NHW mice. (A) Schematic diagram of chemogenetic inhibition of PVN^AVP^ neurons in ovarian HW animals. (B) Representative immunofluorescent images of c-Fos (green) in mCherry-positive PVN^AVP^ neurons (red) post-saline or DCZ treatment in AVP-IRES-Cre mice. Colabeled c-Fos and mCherry (yellow) indicate PVN^AVP^ neuron activation. Notably, DCZ treatment reduced costaining (yellow). (C to H) Behavioral effects with and without DCZ: (C) center time in OFT (**P = 0.0080), (D) SPT consumption (***P = 0.0006), (E) TST immobility (**P = 0.0074), (F) FST immobility (**P = 0.0029), (G) social preference (*P* = 0.5569), and (H) social discrimination (*P = 0.0314). Unpaired *t* test, *n* = 10 for (C) to (H). (I) Chemogenetic activation of PVN^AVP^ neurons in NHW female mice. (J) Representative immunofluorescent images of c-Fos (green) in mCherry-positive PVN^AVP^ neurons (red) post-saline or DCZ treatment in AVP-IRES-Cre mice. Increased costaining (yellow) is observed in the right panel with DCZ treatment. (K) to (P) are similar to (C) to (H). (K) **P = 0.0058, (L) ***P = 0.0004, (M) ***P = 0.0003, (N) ***P = 0.0004, (O) *P* = 0.4004, (P) **P = 0.0024. Unpaired *t* tests, *n* = 12 for (K) to (P).

Given that PVN^AVP^ neurons showed higher activation in the HW mice (Fig. 3), we hypothesized that the increased activity of these neurons might contribute to depressive-like behaviors in HW mice. To test this hypothesis, we employed chemogenetics to selectively manipulate PVN^AVP^ neurons. Specifically, we injected Cre-dependent AAV vectors encoding hM4Di and hM3Dq receptors into the PVN of AVP-IRES-Cre mice, which selectively target AVP neurons (Fig. 4A, B, I, and J). Three weeks later, chemogenetic silencing or activation of PVN^AVP^ neurons was achieved following DCZ administration (Fig. 4B and J). Following DCZ injection, HW mice exhibited significant reductions in depressive-like behaviors. These improvements were characterized by increased time spent in the central zone of the OFT (Fig. 4C), enhanced sucrose preference (Fig. 4D), and decreased immobility time in the TST and FST (Fig. 4E and F), alongside improved social discrimination (Fig. 4H), while social preference remained unchanged (Fig. 4G). Conversely, chemogenetic activation of PVN^AVP^ neurons in NHW mice resulted in exacerbated depressive-like behaviors (Fig. 4K to P). Interestingly, in naive female mice without hormone manipulations, depressive-like behaviors were unaffected by chemogenetic manipulation (Fig. S6). This suggests that hormonal fluctuations are necessary for PVN^AVP^ neurons to influence behaviors. Taken together, PVN^AVP^ neuron activation specifically exacerbates depressive-like behaviors in HW conditions.

### AVP mediates depressive-like behaviors of PVN^AVP^ neurons in HW mice

**Fig. 5.**
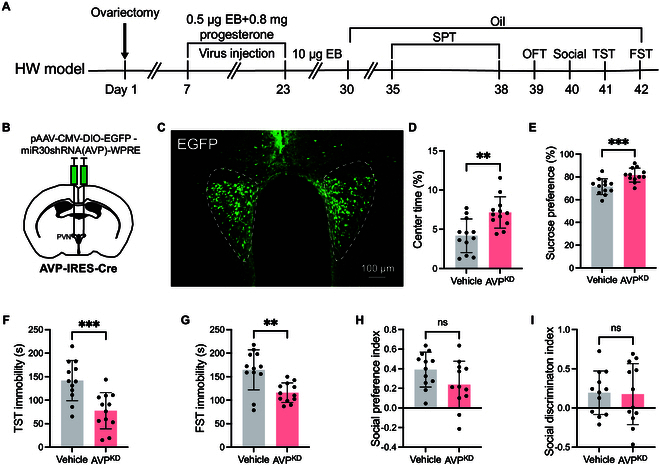
AVP knockdown in the PVN reduces depressive-like behaviors in HW mice. (A) Experimental timeline. (B) Schematic diagram of AAV vector injection into the PVN of AVP-IRES-Cre mice. (C) Representative image of viruses with AVP short hairpin RNA (shRNA) (green) in the PVN. (D to I) Behavioral tests with and without AVP knockdown (KD). (D) Percentage center time in OFT (**P = 0.002). (E) Sucrose preference in SPT (***P = 0.001). (F) TST immobility (***P = 0.0008). (G) FST immobility (**P = 0.0018). (H) Social preference (*P* = 0.0876). (I) Social discrimination (*P* = 0.9041). *n* = 12 for (D) to (I), all comparisons used unpaired *t* tests.

Having established that PVN^AVP^ neurons regulated depressive-like behaviors, we next investigated whether AVP is essential for these behaviors in HW mice. To achieve this, we utilized AAV vectors to knock down AVP expression in PVN^AVP^ neurons (Fig. 5A to C). After 3 weeks, successful down-regulation of AVP was confirmed (Fig. S7). AVP knockdown significantly alleviated depressive-like behaviors, as indicated by increased sucrose preference (Fig. 5E), enhanced center time in the OFT (Fig. 5D), and reduced immobility time in both the TST (Fig. 5F) and FST (Fig. 5G). However, AVP knockdown did not affect social preference (Fig. 5H) or social discrimination (Fig. 5I) in HW mice. These results suggest that AVP release from PVN^AVP^ neurons contributes to depressive-like behaviors in HW mice without influencing social interactions.

### The MPOA-PVN^AVP^ circuit regulates depressive-like behaviors in female mice

**Fig. 6.**
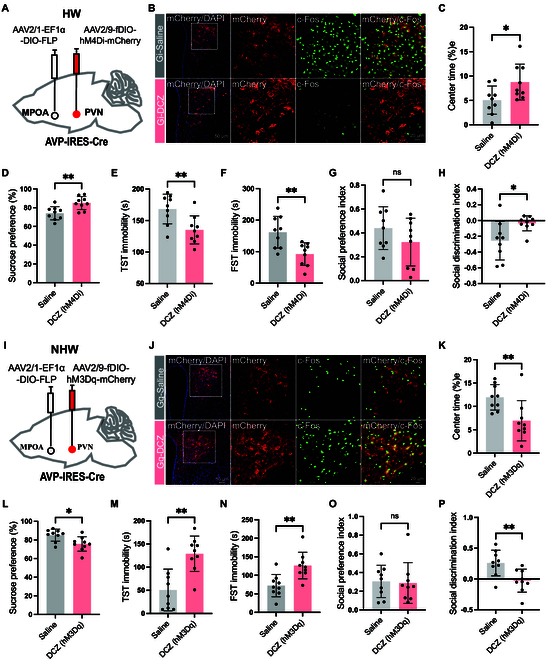
Chemogenetic manipulation of PVN^AVP^ neurons receiving projections from the MPOA modifies depressive-like behaviors in HW and NHW mice. (A) Schematic of chemogenetic inhibition of PVN^AVP^ neurons receiving anterograde input from the MPOA. (B) Representative immunofluorescent images of c-Fos protein expression in mCherry-positive PVN^AVP^ neurons after saline or DCZ treatment in AVP-IRES-Cre mice. (C to H) Behavioral assessments with or without DCZ: (C) center time in the OFT (*P = 0.0304), (D) sucrose preference in the SPT (**P = 0.0044), (E) immobility in the TST (**P = 0.0074), (F) immobility in the FST (**P = 0.0041), (G) social preference in the three-chamber test (*P* = 0.2078), and (H) social discrimination in the three-chamber test (*P = 0.0234). (I) Schematic of viral infection in ovarian NHW mice. (J) Representative immunofluorescent images of c-Fos protein expression in mCherry-positive PVN^AVP^ neurons after saline or DCZ treatment in AVP-IRES-Cre mice. (K to O) Behavioral assessments with and without DCZ in NHW mice: (K) center time in the OFT (**P = 0.0094), (L) SPT consumption (*P = 0.0124), (M) TST immobility (**P = 0.0011), (N) FST immobility (**P = 0.0031), (O) social preference in the three-chamber test (*P* = 0.8564), and (P) social discrimination in the three-chamber test (**P = 0.0073). All statistical tests were unpaired *t* tests, *n* = 9.

Given that PVN^AVP^ neurons regulate depressive-like behaviors in the HW mice (Fig. 4), we hypothesized that the MPOA-PVN^AVP^ circuit might contribute to these behaviors in HW mice. To test this, we employed chemogenetic manipulation by bilaterally injecting AAV-fDIO-hM4Di-mCherry and AAV-fDIO-hM3Dq-mCherry into the PVN, and AAV2/1-EF1α-DIO-FLP into the MPOA of AVP-IRES-Cre mice (Fig. 6A and I). After 3 weeks, chemogenetic silencing or activation of PVN^AVP^ neurons receiving MPOA inputs was induced by DCZ injection (Fig. 6B and J). Chemogenetic inhibition of the PVN^AVP^ neurons receiving MPOA inputs in HW mice reduced depressive-like behaviors, as evidenced by increased center time in the OFT (Fig. 6C), elevated sucrose preference (Fig. 6D), and decreased immobility time in both the TST (Fig. 6E) and FST (Fig. 6F). Social discrimination was also enhanced (Fig. 6H), whereas social preference remained unchanged (Fig. 6G). In contrast, chemogenetic activation of the PVN^AVP^ neurons receiving MPOA inputs in NHW mice exacerbated depressive-like behaviors (Fig. 6K to P). These findings indicate that the MPOA-PVN^AVP^ circuit specifically contributes to depressive-like behaviors in HW mice.

### Mapping of the connections and direct inhibition from MPOA^GABA^ to PVN^AVP^ neurons

**Fig. 7.**
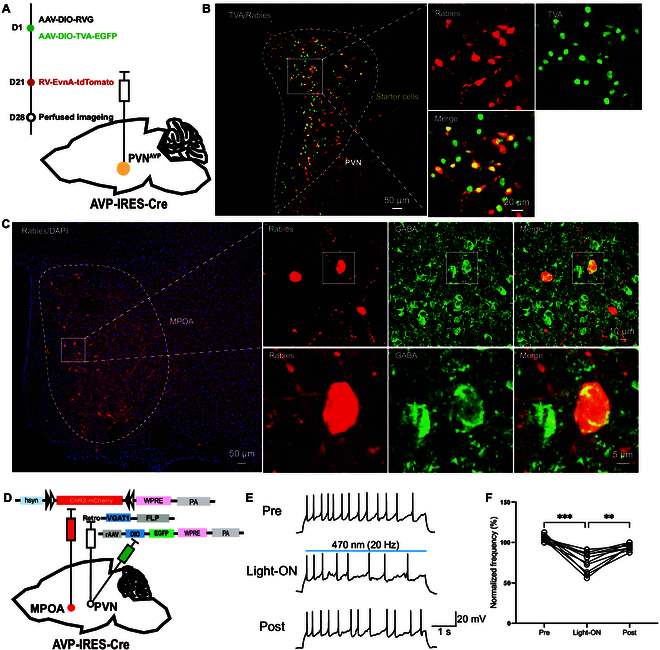
Mapping of the connections and functional connections in the MPOA^GABA^-PVN^AVP^ circuit. (A) Schematic illustration of the injection protocol. On day 1, AAV-EF1α-DIO-TVA-EGFP and AAV-EF1α-DIO-RVG were unilaterally injected into the PVN of AVP-IRES-Cre mice. On day 21, RV-EvnA-tdTomato was injected into the same PVN region to enable retrograde tracing and trans-synaptic labeling. (B) The rabies-tdTomato signal was colocalized with TVA immunofluorescence in the PVN, confirming successful trans-synaptic labeling of neurons projecting to the PVN. (C) Confocal images showing tdTomato (red) and GABA (green) in the MPOA, demonstrating the presence of GABAergic neurons that project to the PVN^AVP^. (D) Schematic diagram illustrating the experimental setup used to assess the functional connection between MPOA GABAergic neurons and PVN AVP neurons. AAV-hsyn-fDIO-hChR2-mCherry was injected into the MPOA, while AAV-VGAT1-FLP was injected into the PVN to enable selective optogenetic activation of MPOA GABAergic terminals. (E) Schematic diagram showing the protocol for blue light stimulation of MPOA GABAergic terminals, with light pulses delivered to the PVN^AVP^ neurons to assess the impact on PVN^AVP^ neuron activity. (F) Representative traces and quantification of the normalized firing frequency of PVN^AVP^ neurons in response to blue light stimulation. Data are presented as normalized frequency before (Pre), during (Light-ON), and after (Post) blue-light treatment. One-way ANOVA, (*F*(1.434, 12.91) = 33.14, ****P < 0.0001). Šídák’s multiple comparisons test for two groups, Pre versus Light-ON (***P = 0.0002); Light-ON versus Post (**P = 0.0012); *n* = 10 cells from 4 mice/group.

Our findings demonstrate that the MPOA GABAergic neurons projecting to the PVN and PVN^AVP^ neurons receiving inputs from the MPOA play opposite roles in regulating depressive-like behaviors (Figs. 2 and 6), suggesting that MPOA GABAergic neurons may exert their effects by inhibiting PVN^AVP^ neurons. To investigate this hypothesis, we employed rabies virus-mediated retrograde trans-synaptic tracing to map monosynaptic inputs onto PVN^AVP^ neurons. Specifically, we stereotaxically injected Cre-dependent AAV helper viruses (rAAV-EF1α-DIO-H2B-EGFP-T2A-TVA and rAAV-EF1α-DIO-ΔRVG) into the unilateral PVN of AVP-IRES-Cre mice (Fig. 7A). After 21 d, RV-CVS-ENVA-N2C(ΔG)-tdTomato was injected into the same PVN site. Seven days later, mice were euthanized for immunohistochemical analysis. Successful trans-synaptic labeling was confirmed by the presence of EGFP-TVA and RV-tdTomato signals in the PVN (Fig. 7B). We identified direct projections to PVN^AVP^ neurons from several ipsilateral brain regions, with prominent inputs originating from the MPOA, bed nucleus of the stria terminalis (BNST), zona incerta (ZI), and PAG (Fig. S8). Notably, many of these MPOA afferent neurons coexpressed GABA (Fig. 7C), indicating that PVN^AVP^ neurons receive monosynaptic inputs from MPOA GABAergic neurons, likely contributing to aversive behavioral outcomes.

To confirm that MPOA^GABA^ neurons exert direct inhibitory effects on PVN^AVP^ neurons, we utilized an ex vivo acute brain slice preparation, allowing for cell-attached recordings from anterogradely labeled PVN^AVP^ neurons. We assessed whether optogenetic activation of MPOA GABAergic afferents could inhibit the activity of PVN^AVP^ neurons in female mice. To achieve this, AAV-EF1α-DIO-EGFP-WPRE was bilaterally injected into PVN to label PVN^AVP^ neurons, while retrograde virus rAAV-VGAT1-FLP-WPRE was injected into the PVN and rAAV-hsyn-fDIO-hCHR2-mCherry-WPRE was injected into the MPOA of AVP-IRES-Cre mice, enabling MPOA GABAergic neurons to express mCherry and ChR2 (Fig. 7D). Three weeks later, brain slices were prepared, and optogenetic activation of ChR2-positive MPOA GABAergic terminals resulted in a significant reduction in the firing frequency of current-evoked action potentials in PVN^AVP^ neurons (Fig. 7E and F). Collectively, these findings demonstrate that MPOA GABAergic neurons establish direct inhibitory synapses onto PVN^AVP^ neurons, thereby modulating their activity and influencing depressive-like behaviors.

## Discussion

**Fig. 8.**
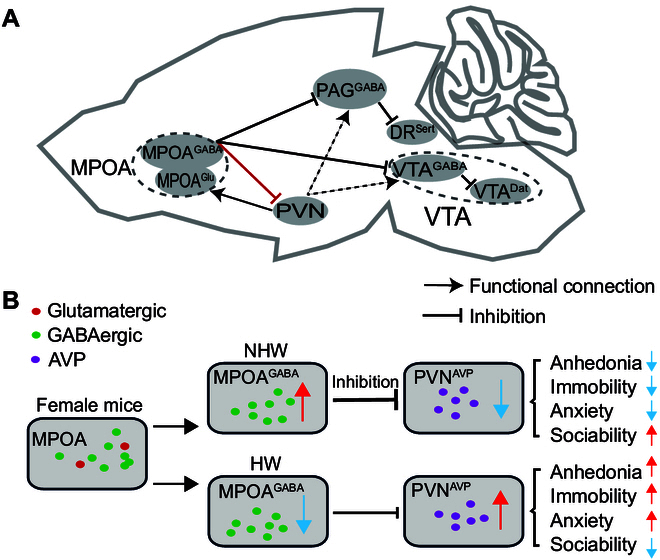
A working model of MPOA-PVN circuit roles in NHW and HW female mice. (A) Based on previous and current findings, MPOA GABAergic neurons project to DR serotonergic neurons or VTA DA neurons via disinhibition of PAG^GABA^ neurons or VTA^GABA^ neurons (previous work) and directly inhibit PVN neurons (current work). PVN neurons may project to PAG/VTA (dashed arrows). Sert, serotonin; Dat, dopamine. (B) Model depicting GABAergic (green) and glutamatergic (red) neurons in the MPOA. Under NHW conditions, elevated activity of MPOA GABAergic neurons (green) may directly inhibit PVN^AVP^ neurons, including AVP-expressing neurons (purple). Conversely, in HW conditions, reduced activity of MPOA GABAergic neurons allows increased activity of PVN^AVP^ neurons, likely involving AVP neurons, which is associated with depressive-like behaviors.

Our study elucidates that the MPOA^GABA^-PVN^AVP^ circuit is a key mediator of depressive-like behaviors, with AVP neurons in the PVN serving a regulatory role. These neurons are modulated by MPOA GABAergic neurons through a monosynaptic inhibitory mechanism (Figs. 3H and I, 7, and 8). These findings provide novel insights into the neural circuits underlying postpartum-related depressive symptoms and highlight potential therapeutic targets for intervention.

### Roles of the MPOA^GABA^-PVN^AVP^ circuit and inhibitory mechanisms of MPOA GABAergic neurons in HW-induced depression

Our findings are consistent with recent research [9] demonstrating that activation of MPOA GABAergic neurons alleviates depressive-like behaviors in HW mice through optogenetics. Using chemogenetics to manipulate these neurons, we reveal that the MPOA^GABA^-PVN^AVP^ circuit is a key mediator of depressive-like behaviors in HW mice. This extends the previous study implicating the role of the MPOA^Glu^-PVN circuit in promoting feeding under different ambient temperatures [25]. However, the projections from MPOA GABAergic neurons to the PVN^AVP^ neurons remained unexplored in HW models until now.

Recent studies have highlighted the involvement of MPOA GABAergic circuits in depressive-like behaviors in HW mice [9]. These studies showed that MPOA GABAergic projections to the VTA predominantly regulate anhedonia, while projections to the PAG primarily modulate immobility [9]. Specifically, MPOA GABAergic projections enhance dopamine (DA) release in the VTA^DA^ neuron by inhibiting VTA GABAergic neurons, as well as boost serotonin release in the dorsal raphe (DR) by inhibiting PAG GABAergic neurons, presumably via a disinhibitory mechanism (see Fig. 8A). Here, we demonstrate that MPOA GABAergic neurons suppress depressive-like behaviors, whereas PVN^AVP^ neurons promote them. Chemogenetic activation of MPOA GABAergic neurons in HW mice reduces c-Fos costaining with AVP in the PVN compared to HW mice without activation (Fig. 3H and I). Supporting these findings, chemogenetic manipulation of PVN^AVP^ neurons (Fig. 4) and AVP knockdown in the PVN (Fig. 5) confirm their direct roles in promoting depressive-like behaviors in HW mice.

Notably, the MPOA and PVN have distinct synaptic architectures, characterized by unique afferent and efferent connections and distinct cell-type distribution [41–43]. We show that PVN^AVP^ neurons receive axonal projections primarily from the MPOA (Fig. S8) and that MPOA GABAergic neurons directly target the PVN^AVP^ neurons (Fig. 7), supporting a functional relationship. Chemogenetic manipulations of the MPOA GABAergic neurons projecting to PVN demonstrated their involvement in the regulation of depressive-like behaviors (Fig. 2), although we cannot rule out a potential contribution of the PAG and VTA neurons, because at least some MPOA neurons project simultaneously to both the PVN and the PAG or the VTA (Fig. S3). However, additional experiments with chemogenetic manipulations of PVN^AVP^ neurons only (Fig. 4) or PVN^AVP^ neurons receiving inputs from the MPOA (Fig. 6), and knocking down AVP in the PVN (Fig. 5) all demonstrated important roles of PVN^AVP^ neurons in modulating depressive-like behaviors in HW mice. Together, we conclude that MPOA GABAergic and PVN^AVP^ neurons play opposing roles in regulating depressive-like behavior (Fig. S2 and Fig. 4). Thus, we propose that the MPOA GABAergic neurons projecting to the PVN^AVP^ neurons regulate HW-induced depressive behaviors via direct inhibition of PVN peptidergic neurons (Figs. 3H and I, 7, and 8), contrasting with the disinhibitory mechanisms previously described for VTA and PAG [9] (Fig. 8A). These results underscore the significance of the MPOA^GABA^-PVN^AVP^ circuit in HW-related depressive-like behaviors and lay a foundation for further exploration of the neural mechanisms underlying complex depressive symptoms.

These findings raise an important question: How does the MPOA^GABA^-PVN^AVP^ circuit integrate with the previously identified MPOA GABAergic projections to the PAG and VTA in HW mice [9]? Interestingly, PVN neurons project to MPOA glutamatergic neurons, which have been shown to promote depressive-like behaviors in the CRS model [44]. Moreover, previous studies suggest that PVN neurons may also project to PAG and VTA [45–47]. Collectively, these findings support 3 possible interpretations (Fig. 8): (a) the MPOA^GABA^-PVN circuit could function as a parallel pathway alongside the MPOA^GABA^-PAG and MPOA^GABA^-VTA circuits, indicating potential redundancy or complementarity in the depression-related neural networks; (b) PVN neurons act as intermediaries, modulating the outputs of MPOA GABAergic neurons to PAG and VTA, thereby influencing depressive-like behaviors; and (c) the PVN-MPOA^Glu^ connection constitutes an additional pathway through which PVN neurons promote depressive-like behavior, forming a microcircuit. However, one study found no significant differences in MPOA^Glu^ neuron activity between HW and NHW mice [9], making the PVN-MPOA^Glu^ circuit a less likely contributor. These possibilities are not mutually exclusive and could all contribute. Thus, our findings expand the current understanding of depression-related neural circuits by highlighting the potential role of the MPOA^GABA^-PVN^AVP^ pathway in HW-associated behaviors. Further research is needed to delineate these circuit interactions and their relevance for depressive disorders.

Intriguingly, MPOA projections to VTA/PAG have also been implicated in depressive-like behaviors in other depression models. In CRS mice, MPOA glutamatergic neurons promote depressive-like behaviors via projections to the PAG and VTA, whereas MPOA GABAergic neurons mediate these behaviors in the social defeat stress mouse model [44], consistent with their observed effects in HW mice [9]. Additionally, in CRS mice, chemogenetic activation of PVN neurons exacerbates depressive-like behaviors, while their suppression attenuates these symptoms [29]. Furthermore, the PVN could act as an upstream modulator of MPOA glutamatergic neurons in driving depressive behaviors in the CRS model [44]. Thus, these findings underscore the importance of the MPOA and PVN in depression across different stress paradigms, reinforcing their potential as therapeutic targets.

### Roles of PVN peptidergic neurons in depression

Neuropeptides are found in both invertebrates [48–52] and vertebrates [53], and they play a critical role in modulating a wide range of behaviors [51,54–60]. The PVN contains various neuropeptide-expressing neurons, including OXT, AVP, and corticotropin-releasing hormone (CRH), each associated with behaviors such as aggression, feeding, sleep, social interaction, and reward [56,57,61]. While the roles of certain PVN neuropeptides have been extensively studied, their involvement in mood regulation remains incompletely understood.

First, OXT plays a complex role in mood-related behaviors. OXT is associated with social memory [62,63], social behavior [64], social reward [65], maternal preference [66], aggression [31], depression [67], and anxiety [68]. In the PVN, OXT neurons not only promote social behaviors [62,63,69] but also enhance anxiety [68] and depressive-like behaviors [67]. Specifically, in male rats and stressed mice (subjected to foot shock, aggression, TST, or restraint), increased activity of OXT neurons in the PVN correlates with enhanced social interaction [62,63,69]. However, long-term stimulation of PVN^OXT^ increases immobility in female mice [67]. In the HW mouse model, modulating PVN oxytocinergic signaling promotes anxiety-like behaviors without affecting sucrose preference [68]. Consistent with this [68], we observed no significant differences in PVN^OXT^ neuron activity between HW and NHW mice (Fig. 3). This suggests that these neurons may not be responsive to depressive-like behaviors induced by HW but could contribute to depression in other mouse models. These findings highlight the complexity of the PVN^OXT^ signaling system in the regulation of behavioral responses.

Second, CRH neurons are implicated in reward-related behaviors [45,70], sleep [71], anxiety [72–74], and depressive-like behaviors [27,28,75]. In the PVN, altering CRH signaling influences depressive-like behaviors in various models, including lipopolysaccharide (LPS)-induced depression [75], chronic social defeat stress (CSDS) [27,28], and chronic unpredictable mild stress (CUMS) models [27,28]. Moreover, the activation of PVN^CRH^ neurons is associated with acute stress-related behaviors, such as digging and grooming, and a reduction in motivated responses for sucrose [70]. These findings suggest that PVN^CRH^ neurons are closely linked to stress-induced depression. Given that PVN^CRH^ neurons do not express ESR1 [76], we hypothesize that ESR1-expressing neurons from the MPOA or other upstream sources may modulate PVN^CRH^ neuron activity to influence HW-induced depressive-like behaviors.

AVP neurons are implicated in various physiological and behavioral processes, including sleep regulation [32], feeding [33], self-grooming [34], nest building [77], social reward processing [35,78,79], stress response [80–82], and aggression [31]. Among these, AVP is particularly prominent in modulating stress response. Our study identifies a crucial role for PVN^AVP^ neurons in mediating depressive-like behaviors in the HW mouse model. We provide initial evidence that activation of PVN^AVP^ neurons correlates with depressive-like behaviors (Fig. 3). Specifically, chemogenetic inhibition of PVN^AVP^ neurons alleviated depressive behaviors in HW mice, while activation of these neurons in NHW mice exacerbated these behaviors (Fig. 4). However, in naïve female mice, activation of PVN^AVP^ neurons had no significant impact on depressive-like behaviors (Fig. S6), consistent with findings that PVN neuron activation contributes to depressive-like behaviors in CRS male mice but not in naive male mice [29]. This suggests that the PVN plays a role in depressive behaviors primarily under conditions of stress or hormonal fluctuations.

Additionally, enhancing PVN^AVP^ neuron activity in normal mice has been linked to increased self-grooming [34] and wakefulness [32] while reducing feeding [33], nest building [77], and social investigation [35]. These findings indicate a broader role of AVP or PVN^AVP^ neurons in mood regulation and motivated behaviors relevant to depression. Importantly, we demonstrate that selectively reducing AVP expression in the PVN of HW mice mitigates depressive behaviors without affecting social discrimination (Fig. 5), suggesting that PVN^AVP^ neurons modulate mood disorders specifically, rather than social behaviors [79]. Our findings are consistent with prior reports that AVP mRNA expression is up-regulated in PVN neurons of rats exposed to stress-induced depression models [36]. Furthermore, AVP knockdown has been associated with increased sucrose preference and reduced immobility in the FST in rats undergoing morphine withdrawal [38]. Pharmacological blockade of the AVP receptor AVPR1b in the basolateral amygdala (BLA) [83] or other brain regions [84,85] has also been shown to alleviate depressive symptoms in rodent models. While these studies collectively support our current findings, our study is the first to demonstrate the pivotal role of AVP in the HW model, potentially mediated through AVPR1b, which has been previously implicated in depressive-like behaviors [83–85]. However, the downstream targets of AVP signaling within the PVN remain unclear, with the BLA being a potential candidate [83]. Future studies are needed to address these important issues. Overall, these findings imply that AVP is a crucial factor in the development and manifestation of depressive behaviors across diverse depression models.

In conclusion, our research reveals that the PVN^AVP^ neurons serve as a downstream target of MPOA GABAergic neurons in the HW mouse model. These neurons play a pivotal role in mediating depressive-like behaviors, with AVP acting as a key regulatory factor. The MPOA^GABA^-PVN^AVP^ circuit may function as a complementary or intermediate pathway alongside the previously identified MPOA^GABA^-VTA and MPOA^GABA^-PAG circuits [9], contributing to the regulation of depressive-like behaviors in the HW model. These findings highlight the MPOA^GABA^-PVN^AVP^ circuit as a critical neural substrate underlying HW-induced depressive behaviors and underscore its potential as a therapeutic for hormone-related mood disorders.

## Materials and Methods

### Animals

Female C57BL/6J mice were purchased from GemPharmatech Co. Ltd., and genetically modified AVP-IRES-Cre and GAD2-IRES-Cre mice were obtained from Shanghai Model Organisms Center Inc. All animals were maintained in a specific pathogen-free (SPF) animal facility, with AVP-IRES-Cre and GAD2-IRES-Cre mice cohabiting in a breeding setup of 1 male and 2 females per cage. Female mice used in this study were between 6 and 12 weeks old. Housing conditions included a 12-h light/dark cycle, with lights on at 9:00 AM and off at 9:00 PM, and unrestricted access to food and water. After ovariectomy, females were housed individually for 1 week to recover before further experimental procedures. All experimental procedures were performed during the light cycle, between 08:00 and 18:00, to minimize the potential effects of circadian rhythm on the results. The experimental protocols were subjected to a rigorous ethics review and were subsequently approved by the Science and Technology Ethics Committee of Nanjing University.

### Establishment of the PPD mouse model

A week post-ovariectomy, each female mouse received daily injections (10:00 AM to 2:00 PM) of 0.05 ml of olive oil containing 0.5 μg of EB (Sigma, E8875) and 0.8 mg of progesterone (Sigma, V900699) for 16 consecutive days. Starting on day 17, mice were injected daily with 0.05 ml of olive oil containing 10 μg of EB for 7 d. Following this regimen, mice were allocated to either an HW group, receiving daily injections of olive oil only, or an NHW group, which continued to receive olive oil containing 10 μg of EB (Fig. S1A). 

### Behavioral tests

#### Open-field test

The OFT (Fig. S1B) was used to assess mouse’s spontaneous locomotion, exploratory behavior, and anxiety levels. Mice were placed in a white test box (25 cm × 25 cm × 50 cm), divided into central and peripheral zones, with movement recorded for 5 min. In the software analysis, the whole area was divided into 3 × 3 grid, defining the central zone as the middle square and the remaining as the peripheral zones. Between tests, the apparatus was cleaned with alcohol and dried.

#### Sucrose preference test

The SPT, illustrated in Fig. S1C, was used to assess anhedonia, a key indicator of depressive-like behavior in mice. To conduct the SPT, mice were first acclimated to the testing environment by providing access to both a 2% sucrose solution and water for a period of 48 h. Following a 16-h period of water deprivation, each cage was equipped with one bottle of sucrose solution and one bottle of water, and the consumption of each fluid was measured over a 24-h period. To control for potential biases in liquid preference, the positions of the bottles were switched after 12 h. The sucrose preference was then calculated as a percentage using the following formula: (volume of sucrose solution consumed/total volume of liquid consumed) × 100%.

#### Tail suspension test

The TST (Fig. S1D) is a classic and rapid method for evaluating depressive-like behavior. The experiment involves suspending mice with their tails, and the animal struggles in this environment, trying to escape the predicament. After efforts to break free prove futile, the animal exhibits intermittent immobility reflective of “behavioral despair”. The test duration was 6 min, during which the animals’ behavior was observed, and the immobility time was specifically measured and recorded during the last 4 min of the test.

#### Forced swim test

The forced swim test (FST), illustrated in Fig. S1E, involved placing mice in a cylindrical tank (12 cm in diameter and 25 cm in height) filled with water at a temperature range of 23 to 25 °C for 6 min. Using specialized software, the immobility time was quantified and recorded during the final 4 min of the test, serving as an indicator of depressive-like behavior in the mice.

#### Social preference and social discrimination tests

The three-chamber social test was conducted in 2 phases: habituation and testing. In the habituation phase, mice explored a three-box apparatus (20 cm × 40 cm × 22 cm) with 2 dividers and an empty cage in each side box. After 10 min, the apparatus was cleaned. During the test phase, a novel conspecific (a mouse that had not been previously encountered) was placed in one of the side cages, while the opposite side cage was left unoccupied. The test mouse’s time spent in social versus non-social areas was recorded for 10 min (Fig. S1F). A second novel mouse was later introduced, and interaction time was recorded as an indicator of social discrimination (Fig. S1G). The social preference index and social discrimination index were calculated using the following formulas:

Social preference index = (Time spent with a mouse − Time spent with an empty cage)/(Time spent with a mouse + Time spent with an empty cage)

Social discrimination index = (Time spent with a novel mouse − Time spent with a familiar mouse)/(Time spent with a novel mouse + Time spent with a familiar mouse)

### Stereotactic surgery

#### Coordinates

PVN: anteroposterior (AP) +0.8 mm, mediolateral (ML) ±0.25 mm, dorsoventral (DV) −5.0 mm; MPOA: AP 0 mm, ML ±0.3 mm, DV 5.2 mm.

#### Viral constructs

For chemogenetic manipulation of the MPOA GABAergic neurons and PVN^AVP^ neurons, rAAV-hSyn-EF1α-hM3D(Gq)-mCherry-WPRE-hGH polyA (5.54 × 10^12^ vg/ml (vg: viral genome), 150 nl for PVN, 200 nl for MPOA) and rAAV-EF1α-DIO-hM4D(Gi)-mCherry-WPRE-hGH-polyA (6.45 × 10^12^ vg/ml, 100 nl for PVN, 150 nl for MPOA) were injected into the MPOA or PVN, respectively. For chemogenetic manipulation of the retrograde MPOA^GABA^ projection from PVN, rAAV-EF1α-DIO-FLP-WPRE-hGH pA (5.50 × 10^12^ vg/ml, 150 nl for PVN), rAAV-hSyn-fDIO-hM4D(Gi)-mCherry-WPRE-hGH polyA (5.27 × 10^12^ vg/ml, 200 nl for MPOA), and rAAV-hSyn-fDIO-hM3D(Gq)-mCherry-WPREs (5.53 × 10^12^ vg/ml, 200 nl for MPOA) were injected into the PVN or MPOA of GAD2-IRES-Cre mice. Three weeks after injecting these viruses, chemogenetic manipulation was performed as follows: Mice harboring hM4D (Gi) or hM3D (Gq) were injected intraperitoneally with DCZ [100 μg/kg, MedChemExpress (MCE)] for 40 min before behavioral assessment.

#### Viral vectors for other experiments

To investigate the role of AVP in PVN, pAAV-CMV-DIO-EGFP-miR30shRNA(NC)-WPRE (7.43 × 10^12^ vg/ml, 400 nl for each site as control) and pAAV-CMV-DIO-EGFP-miR30shRNA(AVP)-WPRE (1.02 × 10^13^ vg/ml, 400 nl for each site) were injected into the PVN of AVP-IRES-Cre mice to knock down AVP in PVN neurons. The shRNA(AVP) target sequence was from a previous study and demonstrated 91% knockdown in vitro [86].

To visualize the MPOA GABAergic neurons projecting to PVN, AAV2/R-EF1α-DIO-FLP-WPRE-hGH pA (5.5 × 10^12^ vg/ml, 150 nl for PVN) and rAAV-hSyn-fDIO-EGFP-WPREs (2.05 × 10^12^ vg/ml, 200 nl for MPOA) were injected into the PVN or MPOA of GAD2-IRES-Cre mice, respectively. The MPOA GABAergic neurons appeared green under fluorescence microscopy.

To locate the upstream brain areas of PVN^AVP^ neurons, rAAV-EF1α-DIO-ΔRVG-WPRE-hGH polyA (5.08 × 10^12^ vg/ml, 150 nl for PVN) and rAAV-EF1α-DIO-H2B-EGFP-T2A-TVA-WPRE-hGH pA (5.52 × 10^12^ vg/ml, 150 nl for PVN) were injected into the PVN of AVP-IRES-Cre mice. Three weeks later, RV-CVS-ENVA-N2C(ΔG)-tdTomato [2 × 10^8^ IFU/ml (IFU: infectious focus unit), 100 nl for PVN] was injected into the same site of the PVN. Positive neurons appeared red under fluorescence microscopy.

To identify whether MPOA GABAergic neurons inhibit PVN^AVP^ neurons, we performed stereotaxic injections of the following viral vectors into AVP-IRES-Cre mice: rAAV-hsyn-fDIO-hCHR2-mCherry-WPRE-hGH polyA (5.78 × 10^12^ vg/ml, 300 nl for MPOA ) was injected into the MPOA, while rAAV-EF1α-DIO-EGFP-WPRE-hGH polyA (5.25 × 10^12^ vg/ml, 200 nl for PVN) and rAAV-VGAT1-FLP-WPRE (5.0 × 10^12^ vg/ml, 200 nl for PVN) were injected into the PVN. Three weeks later, whole-cell patch-clamp recordings were performed to assess the functional connectivity between these neurons.

#### Immunofluorescence

Mice were deeply anesthetized with an intraperitoneal injection of 1% sodium pentobarbital (50 mg/kg) and then perfused with saline, followed by a fixative solution consisting of 4% paraformaldehyde (PFA) in phosphate buffer (PB). The brains were subsequently dehydrated in a series of sucrose solutions, first in 20% sucrose overnight and then in 30% sucrose for an additional 24 h. The brains were then sectioned into 25-μm-thick slices, and the sections were blocked in a solution of phosphate-buffered saline (PBS) containing 1% bovine serum albumin (BSA) and 0.3% Triton X-100. Finally, primary antibodies were applied to the sections overnight at 4 °C. The primary antibodies included mouse anti-AVP (1:500, Sigma, MABN856), guinea pig anti-OXT (1:500, Synaptic Systems, 408004), rabbit anti-c-Fos [1:500, Cell Signaling Technology (CST), 2250], mouse anti-ESR1 (1:100, Santa Cruz Biotechnology, sc-71064), rabbit anti-GAD2 (1:100, Proteintech, 2176-1-AP), and rabbit anti-GABA (1:500, Sigma, A2052). Brain slices were incubated with secondary antibodies after washing 3 times in PBS for 5 min. The secondary antibody included anti-mouse Alexa 488 (1:500, CST, 4408S), anti-rabbit Alexa 488 (1:500, CST, 4412S), anti-mouse Alexa 594 (1:500, Thermo Fisher Scientific, A11005), anti-rabbit Alexa 594 (1:400, Jackson ImmunoResearch, JAC-111-585-003), and anti-guinea pig Alexa 594 (1:400, Jackson ImmunoResearch, JAC-106-585-003).

### CTB retrograde tracing

Mice were subjected to unilateral injection of CTB into the VTA, PAG, and PVN. Specifically, in each mouse, 300 nl of CTB488 (BrainVTA, CTB-01) was injected into the PVN, while an equal volume of CTB555 (BrainVTA, CTB-02) was injected into the VTA or PAG. After a 7-d recovery period, the mice were perfused with PBS followed by 4% PFA. The brains were then carefully dissected and postfixed overnight in the same fixative solution. Subsequently, the brain tissue was sectioned into 30-μm slices. The MPOA neurons labeled with either CTB488 or CTB555 or double-labeled with both CTB488 and CTB555 were quantified.

### In vitro electrophysiology

Mice were deeply anesthetized with an intraperitoneal injection of sodium pentobarbital (50 mg/kg). The mouse brain was then sectioned into 300-μm slices using a Leica vibratome and incubated in ice-cold sucrose-based artificial cerebrospinal fluid (ACSF). The ACSF contained the following components: 124 mM NaCl, 2.5 mM KCl, 1.25 mM NaH₂PO₄, 1.3 mM MgCl₂, 26 mM NaHCO₃, 2 mM CaCl₂, and 10 mM d-glucose. The slices were maintained at 35 °C for 30 min and then equilibrated at room temperature for at least 30 min before recording.

Whole-cell patch-clamp recordings were performed using glass micropipettes (5 to 10 MΩ) filled with an internal solution containing 140 mM K-methylsulfate, 7 mM KCl, 2 mM MgCl₂·6H₂O, 10 mM Hepes, 0.1 mM EGTA, 4 mM Na₂-ATP, and 0.4 mM GTP-Tris, with the pH adjusted to 7.2 using CsOH. PVN^AVP^ neurons were visualized using an Olympus BX51WIFSN microscope (Olympus, Tokyo, Japan). In current clamp mode, the firing frequency of PVN^AVP^ neurons was recorded under stepped holding currents ranging from 10 to 50 pA for 5 s. Data acquisition and analysis were conducted using Clampex and Clampfit 10.7 software (Axon Instruments).

### Statistical analysis

All data are expressed as mean ± SEM. Statistical analyses were conducted using GraphPad Prism 10.0 software, employing one-way or two-way analysis of variance (ANOVA), unpaired Student’s *t* tests, and paired Student’s *t* tests. A *P* value of <0.05 was used to define statistical significance.

## Data Availability

All data needed to evaluate the conclusions in the paper are present in the paper and/or the Supplementary Materials. All the data related to this paper may be requested from the authors.
