# Supplementary material for: The Hypothalamic Medial Preoptic Area–Paraventricular Nucleus Circuit Modulates Depressive-Like Behaviors in a Mouse Model of Postpartum Depression
Source: Research (Wash D C). 2025 May 14;8:0701. doi: 10.34133/research.0701 (PMC12076219; doi:10.34133/research.0701)
Supplement: Supplementary 1 — Supplementary information [file research.0701.f1.docx]

**Supplemental information**

**The Hypothalamic Medial Preoptic Area–Paraventricular Nucleus Circuit Modulates Depressive-Like Behaviors in a Mouse Model of Postpartum Depression**

Ping Fu^1†^, Cui-Ping Liu^1†^, Cheng-Yi Liu^1^,Yan-Chu-Fei Zhang^1^, Ju-Ping Xu^1^, Rui-Ting Mao^1^, Xue-Ying Ding^1^, Fan Li^1^, Yi-Long Zhang^1^, Hai-Long Yang^2^, Jing-Ning Zhu^1^**^*^**, Guo Zhang^1^**^*^**, Jian Jing^1, 3, 4^**^*^**

There is a total of eight supporting figures (Fig. S1-S8).

**
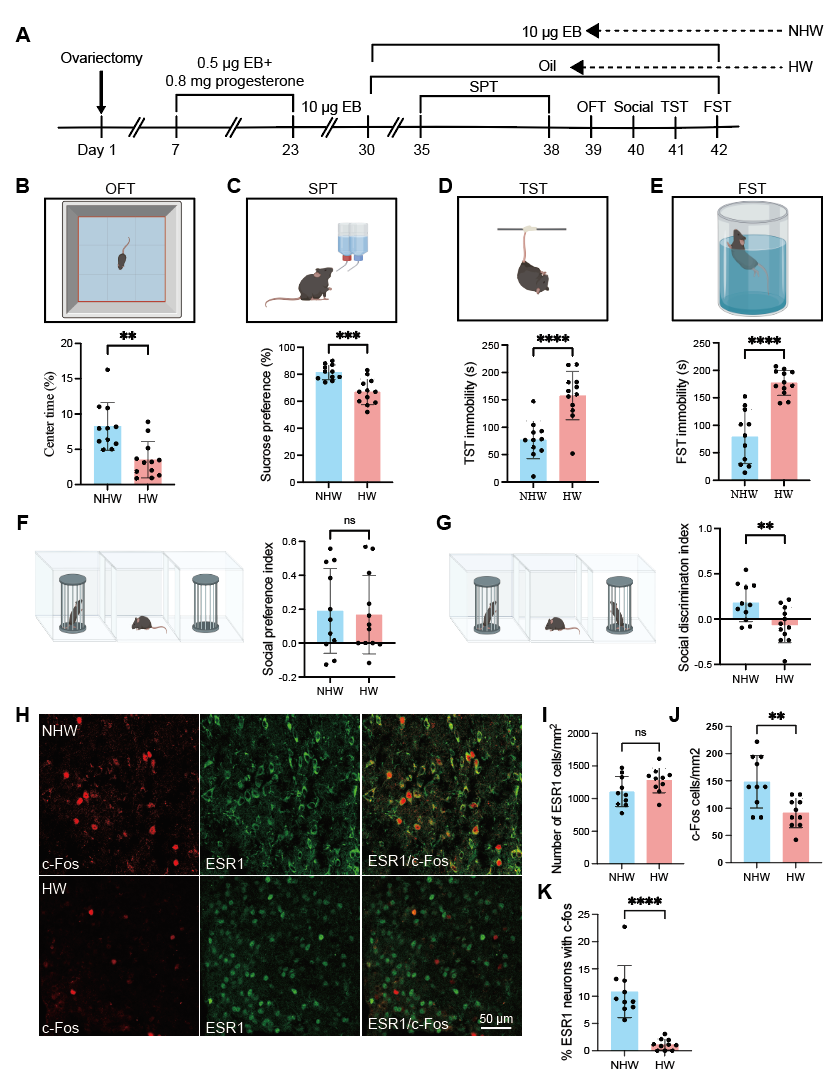
**

**Fig. S1. Model establishment, behavioral test, and the activation of ESR1 in the MPOA of PPD.** (A) Schematic representation of PPD model establishment. EB, β-estrogen. (B) Percentage of center time in the OFT (***P* = 0.0011). (C) Percentage of sucrose preference in the SPT (****P* = 0.0002). (D) Immobility time in the TST (*****P* < 0.0001). (E) Immobility time in FST (*****P* < 0.0001). (F) Social preference in the three-chamber social test (*P* = 0.8224). (G) Social discrimination in the three-chamber social test (***P* = 0.0088). (H) Expression of c-Fos (red) in ESR1 (green) neurons in the MPOA. (I) Quantifying ESR1 neurons in the MPOA (*P* = 0.0878, n = 4). (J) Number of c-Fos-positive cells in the MPOA. Unpaired t-test, NHW vs HW (***P* = 0.0043, n = 4). (K) Proportion of ESR1 neurons expressing c-Fos (*****P* < 0.0001, n = 4). All tests were unpaired t-tests.

**
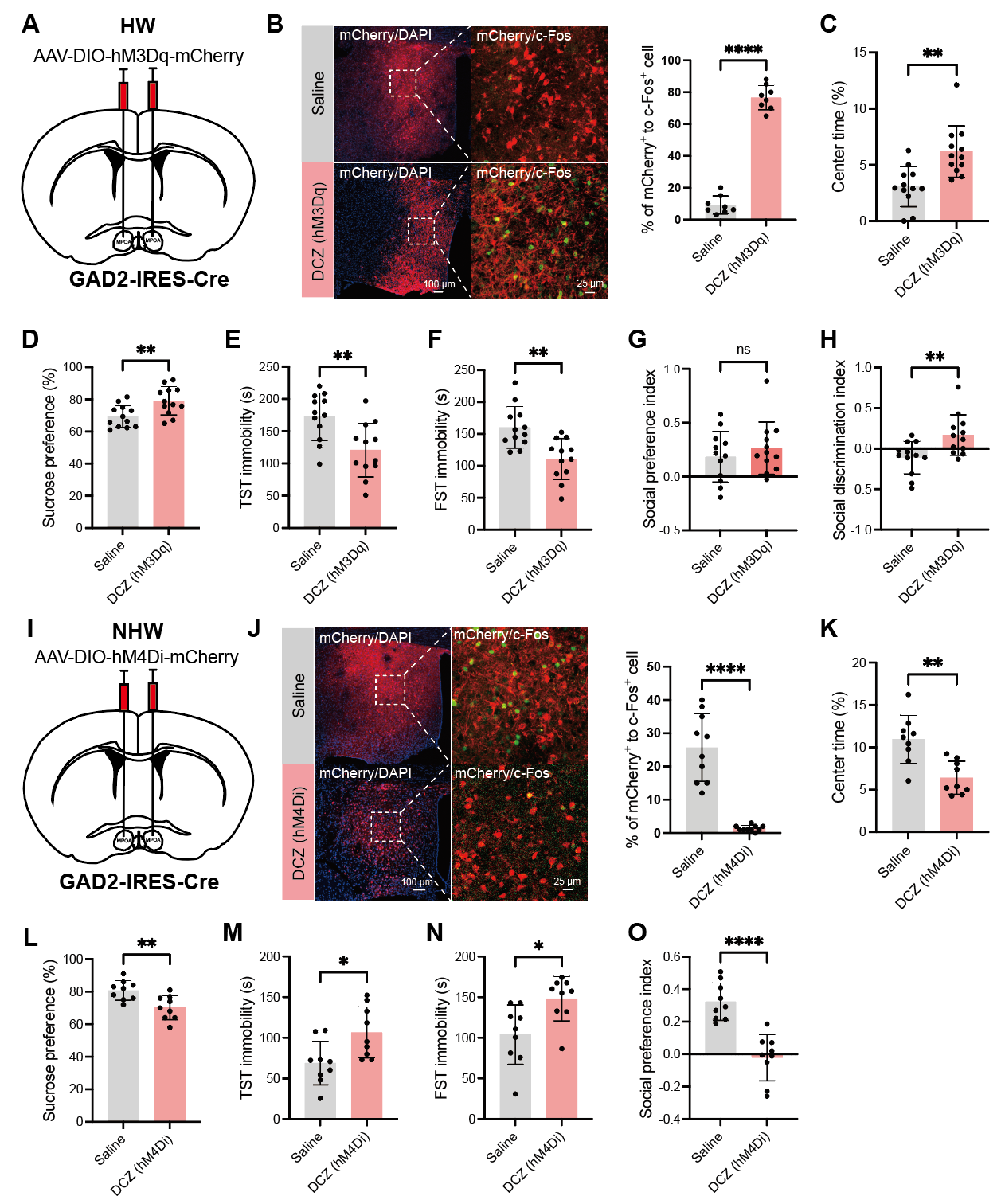
**

**Fig. S2. MPOA GABAergic neurons mediate depressive-like behaviors in HW and NHW mice.** (A) Schematic for chemogenetic activation of MPOA GABAergic neurons in HW mice. (B) Representative immunofluorescent images of c-Fos (Green) in mCherry-positive MPOA GABAergic neurons (red) after saline or DCZ treatment in GAD2-IRES-Cre mice. Co-labeled c-Fos and mCherry (yellow) indicate MPOA GABAergic neuron activation. The right panel shows the co-localization ratio of mCherry/c-Fos after injection of saline or DCZ (*****P* < 0.0001). (C to H) Behavioral measures with and without DCZ: (C) Percentage center time in the OFT (***P* = 0.0011). (D) Sucrose preference in SPT (***P* = 0.0062). (E) TST immobility time (***P* = 0.0040). (F) FST immobility time (***P* = 0.0011). (G) Social preference in the three-chamber social test (*P* = 0.4264). (H) Social discrimination in the three-chamber social test (***P* = 0.0066). n=12 for panels (C) to (H). (I) Schematic for chemogenetic inhibition of MPOA^GABA^ neurons in NHW mice. (J) Representative immunofluorescent images of c-Fos (Green) in mCherry-positive MPOA GABAergic neurons (red) after saline or DCZ treatment in GAD2-IRES-Cre mice. The right image is an example of mCherry and c-Fos (green) expression. The right panel shows the co-localization ratio of mCherry/c-Fos after injection of saline or DCZ (*****P* < 0.0001). Panels (K) to (O) are similar to (C) to (G) but for MPOA GABAergic neuron silencing. (K) ***P* = 0.0012, (L) ***P* = 0.0044, (M) **P* = 0.0145, (N) **P* = 0.0103, (O) *****P* < 0.0001. All tests were unpaired t-tests, n = 9 for panels (K) to (O).

**
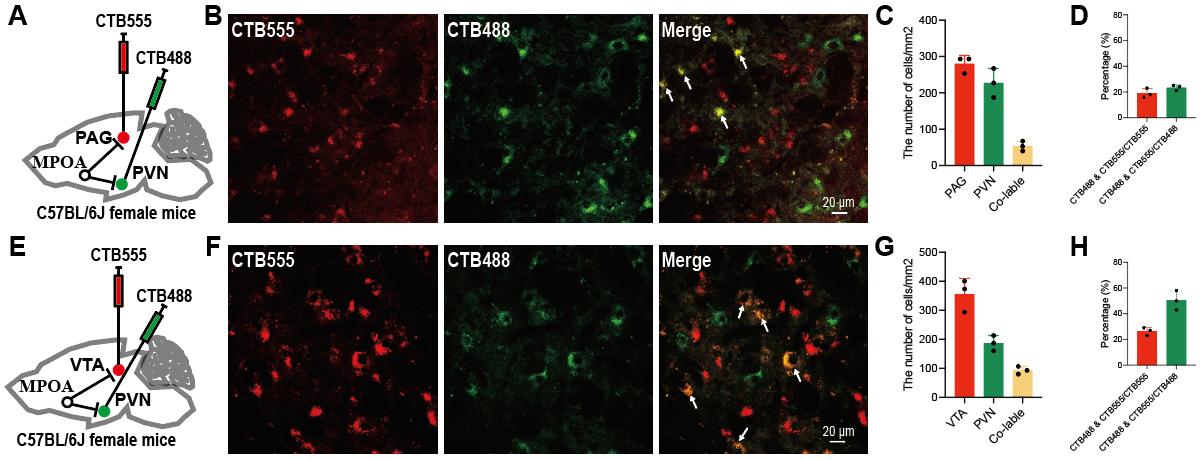
**

**Fig. S3. Dual-color retrograde labeling of MPOA neurons projecting to the PVN and the PAG or the VTA.** (A) Schematic of injections of CTB in the unilateral PAG and the unilateral PVN. (B) Images showing CTB555-labeled (PAG-targeting) and CTB488-labeled (PVN-targeting) neurons in the ipsilateral MPOA, and a merged image of the first two (arrows indicate double-labelled cells). (C) The number of MPOA neurons with CTB labeling per mm^2^ (n = 3). (D) Percentage of double-labeled MPOA neurons relative to PAG (red) or PVN (green) targeting neurons (n = 3). (E) Schematic of injections of CTB in the unilateral VTA and the unilateral PVN. (F) Images showing CTB555-labeled (VTA-targeting) and CTB488-labeled (PVN-targeting) neurons in the ipsilateral MPOA, and a merged image of the first two (arrows indicate double-labelled cells). (G) The number of MPOA neurons with CTB labeling per mm^2^ (n = 3). (H) Percentage of double-labeled MPOA neurons relative to VTA (red) or PVN (green) targeting neurons (n = 3).
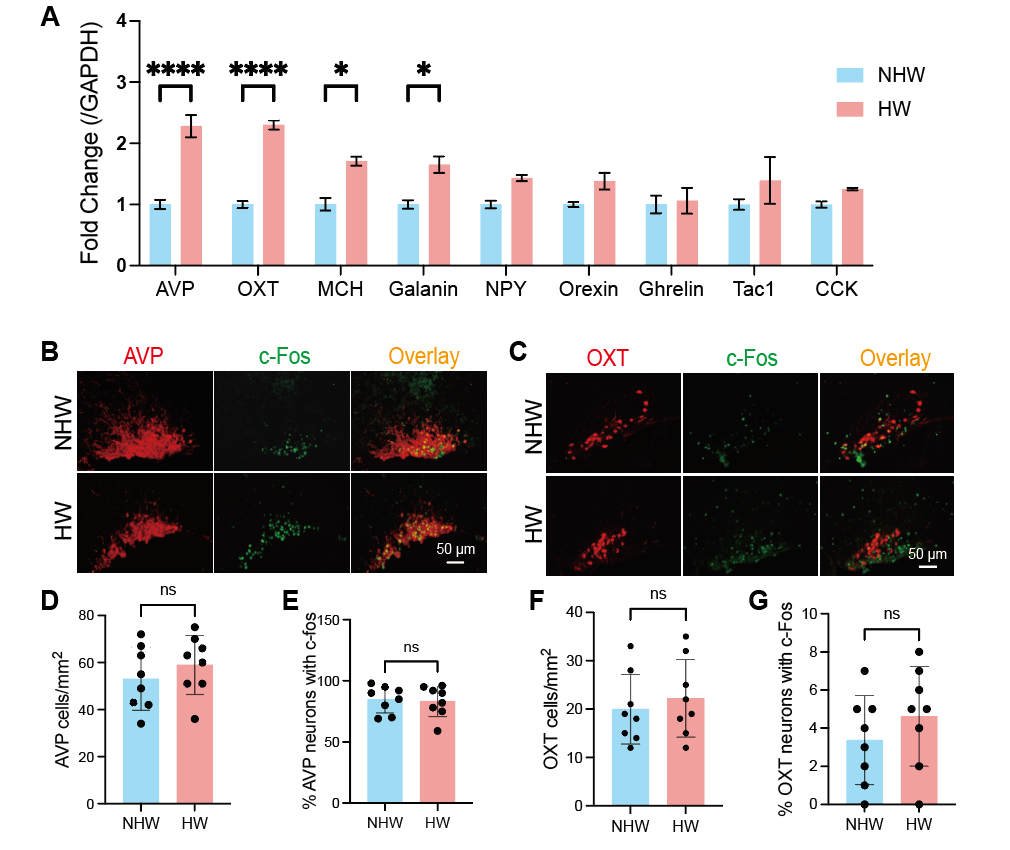


**Fig. S4. Expression of AVP and OXT in NHW and HW mice.** (A) mRNA expression of hypothalamic neuropeptides. Two-way ANOVA, F(8, 46) = 6.02 (*P* < 0.0001). Šídák's multiple comparisons test, NHW vs HW, *****P* < 0.0001 for AVP, n = 6, ****P* < 0.0001 for OXT, n = 5; **P* = 0.0355 for orexin, n = 3. n = 3 for the rest of the peptides. (B to C) Immunofluorescent images of AVP, OXT, and c-Fos (green) co-expression in the supraoptic nucleus (SON). (D) The percentage of AVP neurons co-labeled with c-Fos (*P* = 0.8034, n = 4), unpaired t-test. (E) AVP neurons in SON (*P* = 0.3807, n = 4), unpaired t-test. (F) OXT neurons in SON (*P* = 0.5648, n = 4), unpaired t-test. (G) The proportion of OXT (red) neurons with HW-induced c-Fos (*P* = 0.1636, n = 4), unpaired t-test. MCH: Melanin-concentrating hormone; NPY: Neuropeptide Y; Tac1: Tachykinin 1; CCK: Cholecystokinin.

**
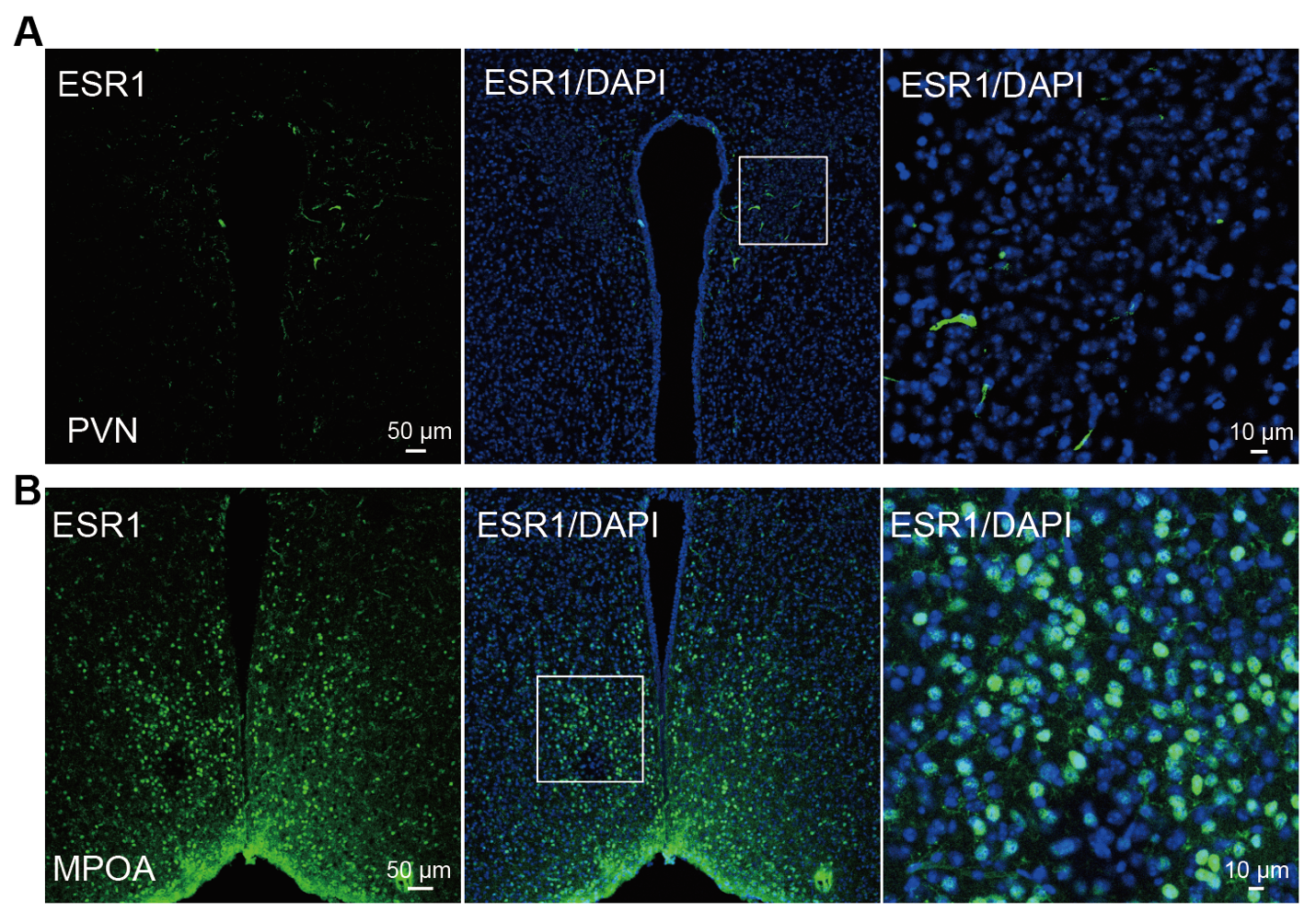
**

**Fig. S5 Absence of ESR1 expression in the PVN.** (A) Representative image showing ESR1 expression (green) in the PVN. (B) Representative image showing ESR1 expression (green) in the MPOA (as control) in female mice. Enlargement (right image, dashed square in the middle) shows details.

**
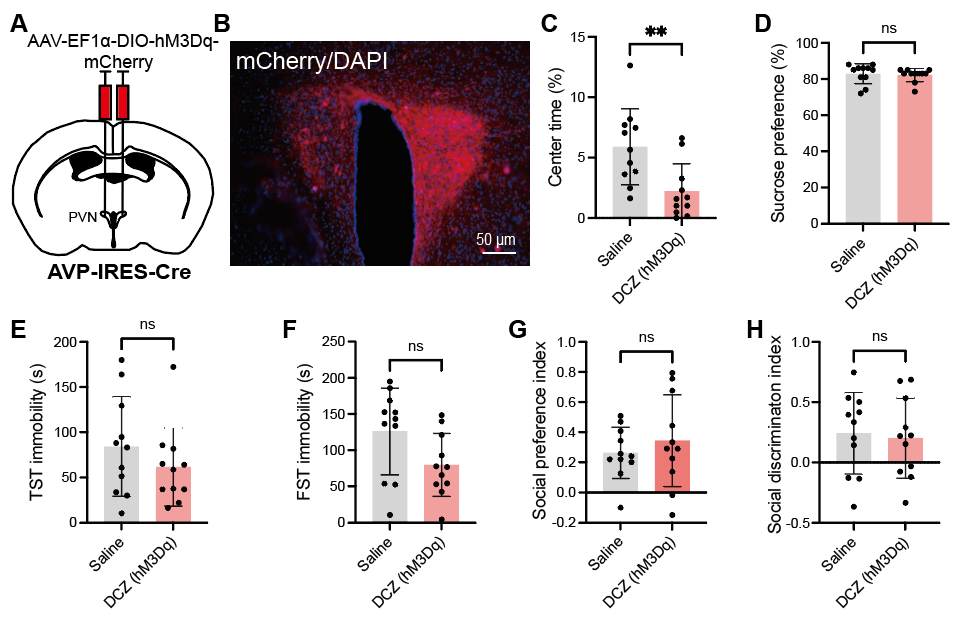
Fig. S6. Activation of PVN^AVP^ neurons in naive female mice without any treatment.** (A) Schematic for selective activation of PVN^AVP^ neurons. (B) Representative image showing AAV-DIO-hM3Dq (red) and DAPI (blue) expression in the PVN. (C to H) Behavioral assessments: (C) Percentage center time in the OFT (***P* = 0.005). (D) Sucrose preference in SPT (*P* = 0.7151). (E to F) TST and FST immobility time, *P* = 0.2973 for TST, *P* = 0.0518 for FST. (G) Social preference and (H) social discrimination in the three-chamber social test, *P* = 0.4519 for social preference, *P* = 0.7884 for social discrimination. n = 11, unpaired t-test for all statistics.

**
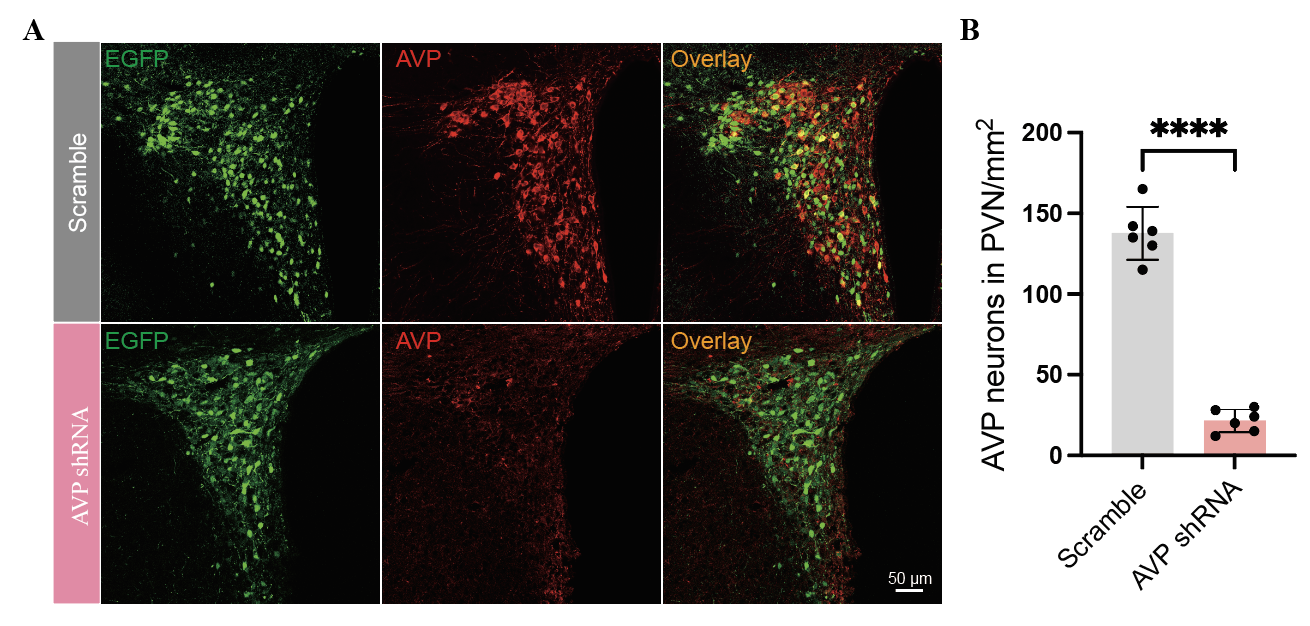
**

**Fig. S7. AVP knockdown in the PVN of HW mice.** (A) Representative images show AVP immunoreactivity (red) reduction with AVP shRNA (bottom) compared with Scramble RNA (Top) in the PVN. EGFP represents infection with viruses conjugated with either scramble RNA (Top) or shRNA (Bottom) in AVP-IRES-Cre mice. Right panels: Co-expression (yellow) of EGFP (green) and AVP (red) indicates knockdown efficiency, with fewer yellow cells indicating effective knockdown. (B) Analysis of AVP-immunoreactive cells in PVN with AVP shRNA (vs. Scramble), unpaired t-tests (*****P* ＜ 0.0001).

**
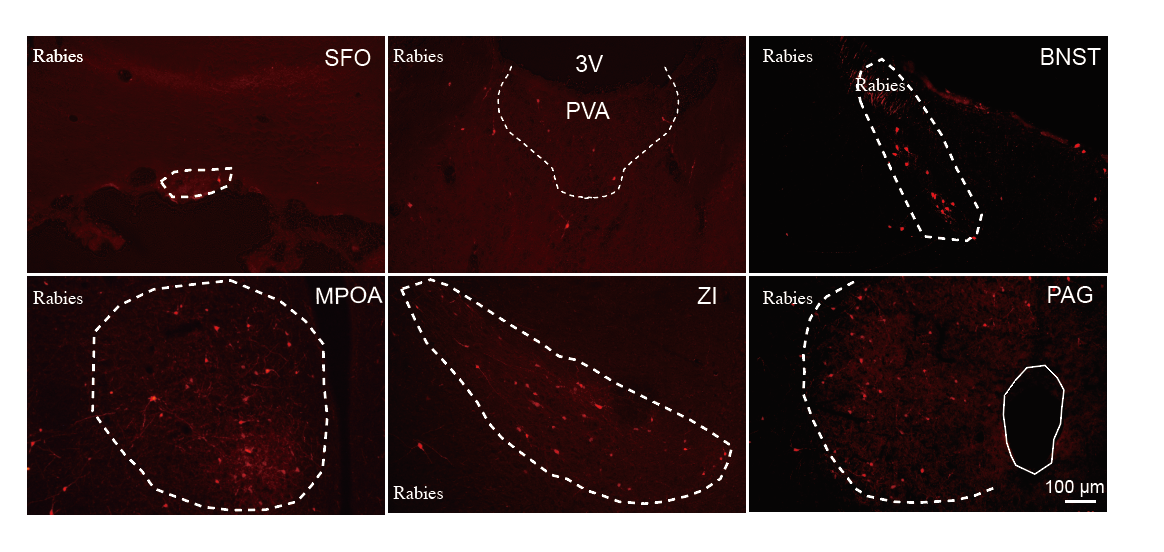
**

**Fig. S8. Monosynaptic inputs to PVN^AVP^ neurons (Retrograde tracing).** Representative retrogradely labeled neurons in various brain regions: SFO, PVA, BNST, MPOA, ZI, PAG. SFO, subfornical organ; 3V, 3rd ventricle; PVA, paraventricular nucleus of thalamus; BNST, bed nucleus of the stria terminalis; MPOA, medial preoptic area; ZI, Zona incerta; PAG, periaqueductal grey.
